# Changes in Default Alarm Settings and Standard In-Service are Insufficient to Improve Alarm Fatigue in an Intensive Care Unit: A Pilot Project

**DOI:** 10.2196/humanfactors.5098

**Published:** 2016-01-11

**Authors:** Azizeh Khaled Sowan, Tiffany Michelle Gomez, Albert Fajardo Tarriela, Charles Calhoun Reed, Bruce Michael Paper

**Affiliations:** ^1^ Department of Health Restoration and Care Systems Management School of Nursing University of Texas Health Science Center at San Antonio San Antonio, TX United States; ^2^ Center for Excellence in Patient Care University Health System San Antonio, TX United States; ^3^ University Health System Transplant Cardiac Intensive Care Unit (ICU) San Antonio, TX United States; ^4^ Office of Nursing Research School of Nursing University of Texas Health Science Center at San Antonio San Antonio, TX United States

**Keywords:** cardiac monitors, default alarm settings, alarm fatigue, intensive care unit, nursing, in-service, survey

## Abstract

**Background:**

Clinical alarm systems safety is a national concern, specifically in intensive care units (ICUs) where alarm rates are known to be the highest. Interventional projects that examined the effect of changing default alarm settings on overall alarm rate and on clinicians’ attitudes and practices toward clinical alarms and alarm fatigue are scarce.

**Objective:**

To examine if (1) a change in default alarm settings of the cardiac monitors and (2) in-service nursing education on cardiac monitor use in an ICU would result in reducing alarm rate and in improving nurses’ attitudes and practices toward clinical alarms.

**Methods:**

This quality improvement project took place in a 20-bed transplant/cardiac ICU with a total of 39 nurses. We implemented a unit-wide change of default alarm settings involving 17 parameters of the cardiac monitors. All nurses received an in-service education on monitor use. Alarm data were collected from the audit log of the cardiac monitors 10 weeks before and 10 weeks after the change in monitors’ parameters. Nurses’ attitudes and practices toward clinical alarms were measured using the Healthcare Technology Foundation National Clinical Alarms Survey, pre- and postintervention.

**Results:**

Alarm rate was 87.86 alarms/patient day (a total of 64,500 alarms) at the preintervention period compared to 59.18 alarms/patient day (49,319 alarms) postintervention (*P*=.01). At baseline, Arterial Blood Pressure (ABP), Pair Premature Ventricular Contractions (PVCs), and Peripheral Capillary Oxygen Saturation (SpO2) alarms were the highest. ABP and SpO2 alarms remained among the top three at the postproject period. Out of the 39 ICU nurses, 24 (62%) provided complete pre- and postproject survey questionnaires. Compared to the preintervention survey, no remarkable changes in the postproject period were reported in nurses’ attitudes. Themes in the narrative data were related to poor usability of cardiac monitors and the frequent alarms. The data showed great variation among nurses in terms of changing alarm parameters and frequency of replacing patients' electrodes. Despite the in-service, 50% (12/24) of the nurses specified their need for more training on cardiac monitors in the postproject period.

**Conclusions:**

Changing default alarm settings and standard in-service education on cardiac monitor use are insufficient to improve alarm systems safety. Alarm management in ICUs is very complex, involving alarm management practices by clinicians, availability of unit policies and procedures, unit layout, complexity and usability of monitoring devices, and adequacy of training on system use. The complexity of the newer monitoring systems requires urgent usability testing and multidimensional interventions to improve alarm systems safety and to attain the Joint Commission National Patient Safety Goal on alarm systems safety in critical care units.

## Introduction

Bedside physiologic monitors are equipped with alarm systems for patient safety and appropriate functionality. Nevertheless, the problematic high volume of false and clinically insignificant nonactionable true positive alarms—up to 99.4%—results in clinicians’ failure to appropriately respond to alarms signaled from monitoring devices [1-5]. Clinicians become overwhelmed and desensitized with the number of alarms, a phenomenon known as alarm fatigue. Alarm fatigue leads to different forms of unsafe workarounds, including a delayed response, disabling alarms, turning the volume to inaudible, or adjusting alarms’ settings to hazardous limits, all of which can result in missing lethal alarms. The Joint Commission (JC), which accredits and certifies health care organizations and programs in the United States, attributed alarm-related incidents and deaths to alarm fatigue and issued a 2014 National Patient Safety Goal to improve the safety of clinical alarm systems [6].

Alarm safety is a priority in intensive care units (ICUs) where alarm rates are known to be the highest [7,8]. Adjusting default alarm settings and staff education on alarm management are two strategies recommended by safety and professional organizations to reduce the number of false alarms and alarm fatigue [9-11]; however, most research on alarm safety is observational. Interventional projects that examined the effect of changing default alarm settings on overall alarm rate and on clinicians’ attitudes and practices toward clinical alarms and alarm fatigue are scarce [12,13]. To address these gaps in the literature, this project aims to examine if (1) a change in default alarm settings of the cardiac monitors and (2) in-service nursing education on monitor use in an ICU would result in reducing alarm rate and improving nurses’ attitudes and practices toward clinical alarms.

## Methods

### Design, Setting, and Sample

After obtaining Institutional Review Board (IRB) approval, this quality improvement project took place in a 20-bed transplant/cardiac ICU located at a university teaching Magnet hospital in the Southwest of the United States. The unit has 39 nurses and an average annual admission of 1500 patients. In April 2014, the unit went through three simultaneous changes, including a move to a new tower and the deployment of new bedside cardiac monitors (Philips IntelliVue MX800) and Cisco phones. The unit is an E shape and has three central nursing stations equipped with cardiac monitors (Philips IntelliVue Information Center iX) with no dedicated monitor watchers. Typical training on new medical devices includes a few hours' demonstration on the appropriate use of devices by the company representative and/or the unit nurse educators. Nurse educators also provide “if needed” support on equipment use. The interventions took place 2.5 months after the move to the new tower and the use of the new cardiac monitors.

Philips IntelliVue MX800 and the Information Center iX cardiac monitors are equipped with complex information systems with tens of main menus and as many submenus, keys, buttons, and icons to facilitate patient data surveillance and management. The monitors are operated using an interactive touch screen, a mouse and a keyboard, or a remote control. The monitors are also capable of complex functions such as lab data integration, drug calculations, guiding care through embedded clinical protocols, issuing reports and strips, presenting trended alarm data, and displaying hundreds of different alarm messages.

### The Interventions

The interventions included (1) changing default settings of some parameters on the cardiac monitors and (2) re-educating transplant/cardiac ICU bedside nurses on the appropriate use of the monitors. Default settings were changed based on scientific clinical rational and recent evidence [7,12-14]. Parameters involved in the change are presented in Table 1. Change methods included the following:

1. Limit tightening.

2. Limit increase.

3. Changing the source of alarm detection to enhance alarm reading.

4. Changing the measurement mode in order to capture real conditions from different measurement sources. Measurement modes were changed from “One Source” to “Auto” (eg, System Pulse) and from “Auto” to “Enhanced” (eg, Asystole). “Auto” and “Enhanced” modes allow the monitor to look for an alternate heart rate source, such as the pulse oximeter or the arterial line if it cannot pick up a rhythm from the electrocardiogram (ECG) leads.

5. Alarm delay by increasing the period from alarm detection to announcement.

6. Disabling alarms, for example, Noninvasive Blood Pressure (NBP) Done Tone and Atrial Fibrillation (AFIB). NBP Done Tone is a nonactionable alarm announced automatically by the monitor after measuring the patient blood pressure. The AFIB alarm was disabled because it is also captured by the Irregular Heart Rate alarm. The definitions of alarm events involved in the changes are presented in Multimedia Appendix 1; some of the definitions were adapted from the IntelliVue Information Center iX Guide [15].

Changes were directed toward decreasing the number of false alarms and increasing monitoring safety. For example, although tightening the Premature Ventricular Contractions (PVCs)/minute from 10 bpm to 6 bpm is expected to increase alarm events of this parameter, this tightening was necessary for safety purposes because all other PVC-related alarms, such as Run PVCs, Pair PVCs, Bigeminy, Trigeminy, and Multiform PVCs, were disabled. Similarly, although the limit of ExtremeTachy was increased to decrease the number of false alarms, TachyClamp was tightened for safer monitoring.

7. Volume adjustment including (1) decreasing the volume of yellow alarms with moderate priority, for example, Heart Rate (HR), and (2) increasing the volume of high-priority red alarms, for example, Desaturation. Changes in alarm volume are not expected to directly affect alarm rates, but rather to focus the nurse's attention on actionable high-priority alarms for safety purposes.

All parameters involved in the change are amenable to adjustments by clinicians except for TachyClamp and ExtremeTachy, which are considered hard stops for system safety and can be adjusted only by Philips representatives (see Table 1).

The nursing unit educators conducted roaming individual in-service sessions. Educational sessions included all nurses in the unit and focused on assessment of monitor parameters, customizing parameters to be patient specific, steps of changing alarm parameters, steps of printing alarm parameters, relearning arrhythmias and changing lead analysis, and troubleshooting common alarming problems (eg, silencing alarms of monitors not connected to patients).

### Procedure and Instrumentation

A team of three expert transplant/cardiac ICU nurses and a Philips representative created the list of proposed changes in parameters. This list then went through a review and approval process by all transplant/cardiac ICU physicians, nurse directors, educators, managers, and bedside nurses. The list of approved changes is presented in Table 1. After approval and before implementing any changes to bedside monitors, we invited all transplant/cardiac ICU nurses to complete a survey about nurses’ attitudes and practices toward clinical alarms using an adapted version of the Healthcare Technology Foundation (HTF) National Clinical Alarms Survey [5]. A detailed description of the survey, the adaptation process, and results of the preintervention survey are presented elsewhere [16]. The postintervention survey includes three sections: (1) demographics, (2) 22 items measured on a 5-point Likert scale of agreement measuring nurses’ attitudes toward clinical alarms followed by a free-text comment area, and (3) a rank section of nine items describing issues that threaten alarm recognition and response when using the cardiac monitors. The survey was followed by three additional questions related to (1) frequency of changing alarm parameters, (2) frequency of changing electrodes, and (3) adequacy of the training received on cardiac monitors.

After collecting the preintervention surveys, a Philips representative completed a unit-wide change to all monitors based on the approved list on July 1, 2014. This change was also communicated through emails, shift reports, huddles, and meetings to all transplant/cardiac ICU nurses and physicians. The unit in-service education started right after the changes in monitors’ parameters and lasted for approximately two weeks. After that, an invitation to complete the postintervention survey via SurveyMonkey went out to all nurses using individual emails that included the same ID number used in the preintervention survey. Two email reminders were sent to nonrespondents to enhance the response rate.

Alarm events were measured by retrieving the audit log of the cardiac monitors from the database of the central-station monitors for 10 weeks before and 10 weeks after implementing the changes in parameters. The audit log is a chronological record of all alarm events logged by the bedside cardiac monitors.

### Data Analysis

Nurse characteristics, alarm rate, and attitude toward clinical alarms were described using descriptive statistics. *Z* tests were used to measure the difference in alarm rates per patient day between the preproject and postproject periods. The change in nurses’ attitudes toward clinical alarms was described using a percent change. *t* tests for paired data were used to analyze the difference in mean scores of the ranks assigned to the nine issues affecting alarm recognition (section 3 in the survey) between the preproject and postproject periods.

**Table 1:** Changes in default settings of the cardiac monitors at the transplant/cardiac intensive care unit.

Type of change	Parameter	Default setting	Changed to...
**Limit tightening**			
	PVCs^a^/minute	10 bpm	6 bpm
	TachyClamp^b^	200 bpm	180 bpm
Limit increase	ExtremeTachy^b^	20 bpm > Heart Rate High Limit	40 bpm > Heart Rate High Limit
**Changing the source of alarm detection**			
	ABP^c^	Source: Systolic	Source: Systolic and Mean
	NBP^d^	Source: Systolic and Mean	Source: Systolic
**Changing the measurement mode**			
	System Pulse^e^	SpO2^f^	Auto (from ABP, SpO2, etc)
	Asystole	Standard	Enhanced
Alarm delay	SpO2: Average^g^	No	Yes: 10 seconds
**Disabling alarms**			
	ST^h^ Analysis^i^	On	Off
	Run PVCs	On	Off
	Pair PVCs	On	Off
	Bigeminy PVCs	On	Off
	Trigeminy PVCs	On	Off
	Multiform PVCs	On	Off
	Pause	On	Off
	Missed Beat	On	Off
	AFIB^j^	On	Off
	NBP Done Tone	On	Off
Decrease alarm volume	Yellow Alarm Volume	5	3
Increase alarm volume	Red Alarm Volume	+0	+2

^a^PVC: premature ventricular contraction.

^b^These alarms are not amenable to change by clinicians. All other alarms can be customized by clinicians based on the patient condition.

^c^ABP: arterial blood pressure.

^d^NBP: noninvasive blood pressure.

^e^If the peripheral capillary oxygen saturation (SpO2) had a poor waveform, the pulse from the pleth would not pick up and would therefore alarm. Changing to Auto allows the monitor to detect a pulse from other sources before alarming.

^f^SpO2: peripheral capillary oxygen saturation.

^g^SpO2 will be averaged over 10 seconds to determine a value instead of alarming the second the SpO2 drops below the limit. The nurse can also manually increase this to 20 or 30 seconds.

^h^ST: ST segment in the electrocardiogram.

^i^The ST Analysis alarm was disabled but should be turned on for all interventional cardiology cases (eg, require cardiac catheterization) or acute coronary syndrome patients. For these specific patients, the original limit of +/-2.0 mm should be tightened to +/- 1.6 mm as per physicians’ requests.

^j^AFIB: atrial fibrillation.

## Results

### Nurse Characteristics

Out of the 39 transplant/cardiac ICU nurses who responded to the preintervention survey, 24 (62%) returned completed responses in the postintervention period. General characteristics of the 39 ICU nurses are described elsewhere [16]. The majority of the 24 nurse respondents were female (15/24, 63%) and worked full time (19/24, 79%). Almost half were 30-50 years old (13/24, 54%) and the other majority were less than 30 years old (10/24, 42%). Although 46% (11/24) reported having more than 5 years of nursing experience, 79% (19/24) reported having less than or equal to 5 years of transplant/cardiac ICU experience. Chi-square tests for correlation revealed no significant differences between the 24 nurse respondents and the total 39 transplant/cardiac ICU nurses on age, gender, employment status, or total years of nursing or ICU experiences (*P*>.10).

### Alarm Rate

Table 2 shows the number of alarms, their specific types, and difference in alarm rates per patient day for the parameters targeted in the change between the two project periods. The audit log recorded a total of 64,500 alarms at the preproject period and 49,319 at the postproject period. At baseline, Arterial Blood Pressure (ABP), Pair PVC, and Peripheral Capillary Oxygen Saturation (SpO2) alarms were the highest. ABP and SpO2 alarms remained among the top three at the postproject period. Although we disabled ten parameters (see Table 1), the data showed incomplete elimination of these alarms (see Table 2). We investigated in order to check if these alarms were activated by nurses or if they were missed from the change and discovered that one of our bedside monitors was missed from the unit change. If that monitor had been included in the change, it would have further eliminated 130 alarms (0.16 alarms/patient day) in the postproject period. These alarms included 13 Pair PVCs, 3 Multiform PVCs, 82 Missed Beat alarms, 23 Asystole alarms, and 9 ST alarms. Although we disabled the NBP Done Tone alarm, the audit log does not record this alarm. Therefore, the difference in alarm rates between the two project periods excludes the rate of that specific alarm. Using *Z* tests, the difference in proportions of alarm events (87.86 vs 59.18 alarms/patient day) between the two project periods was significant (*P*=.01), with a 24% reduction of total alarms.

### Survey Results

Although all 39 nurses responded to the preintervention survey [16], we only analyzed the results of the paired sample of nurses who provided complete responses in the preproject and postproject periods (n=24).

#### Nurses’ Attitudes and Practices Toward Clinical Alarms

The internal consistency reliability of the 22-item scale that measured attitude toward alarms using Cronbach alpha was high (.72-.75 for the pretest and post-test, respectively). Table 3 displays percentages of the 24 nurses who agreed or strongly agreed with the statements that measured attitudes toward clinical alarms and the percent change. Almost all nurses agreed/strongly agreed that nuisance alarms are frequent, disrupt patient care, and reduce trust in alarms causing caregivers to disable them (items 1, 2, and 3). Major issues threatening alarm recognition and response according to the majority of nurses in the two project periods were related to the confusion in locating the alarming device (item 4), unit layout (item 8), inadequacy of alarm systems to alert nurses of changes in patients' conditions (item 18), the lack of clinical policies and procedures on alarm management (item 21), and the inability of the newer monitoring systems to solve alarm problems (item 22). The majority of nurses were in favor of using smart alarms and central alarm management staff, and the integration of alarms to wireless devices (items 5, 6, 7, and 9).

The positive changes at the postproject period were related to the requirement to document alarm settings, the distinct outputs of medical devices, effective policies to manage alarms, and the ability of the newer systems to solve alarm problems (items 17, 20, 21, and 22). However, in this project, a positive or negative change in attitude on an item was considered clinically meaningful only if reported by at least one-third of nurses (ie, 8 nurses). Table 3 shows that the number of nurses with a change in attitude in the postproject period ranged from 0 to 6 nurses, therefore no major changes in attitude were reported.

**Table 2:** Difference in alarm rates between the preproject and postproject periods.

Alarm condition		Preproject period		Postproject period	
		Number of alarms	Total alarm rate/patient day	Number of alarms	Total alarm rate/patient day
**ABP** ^a^					
	Total	27,930	38.05	28,049	33.67
	ABPs^b^ (systolic)	13,776		14,726	
	ABPm^c^ (mean)	13,548		12,895	
	ABP disconnect	606		428	
Pair PVCs^d,e^		8305	11.31	164	0.19
**SpO2** ^f^					
	Total	7079	9.64	8290	9.95
	SpO2	6741		7858	
	SpO2r^g^ (right)	338		323	
	SpO2l^h^ (left)	0		109	
Multiform PVCs^e^		5865	7.99	19	0.02
**NBP** ^i^					
	Total	3686	5.02	3976	4.77
	NBPm^j^ (mean)	1847		43	
	NBPs^k^ (systolic)	1837		3933	
	NBPd^l^ (diastolic)	2		0	
PVCs/min		3233	4.40	5330	6.39
Run PVCs high^e^		2155	2.94	23	0.03
ST^e^		1851	2.52	2609	3.13
**AFIB** ^**e,m**^					
	Total	1481	2.02	32	0.04
	AFIB	990		26	
	End AFIB^n^	491		6	
Pause^e^		1086	1.48	8	0.01
Missed Beat^e^		873	1.19	89	0.11
Asystole		323	0.44	565	0.68
**Tachy** ^o^					
	Total	292	0.39	153	0.18
	Tachy	273		153	
	Tachy/p^p^ (tachycardia p wave)	19		0	
Vent^q^ Bigeminy^e^		234	0.32	7	0
Vent Trigeminy^e^		79	0.11	0	0
Pulse		28	0.04	5	0.01
Total		64,500	87.86	49,319	59.18

^a^ABP: arterial blood pressure.

^b^ABPs: arterial blood pressure systolic.

^c^ABPm: arterial blood pressure mean.

^d^PVC: premature ventricular contraction.

^e^These are the alarms that we disabled.

^f^SpO2: peripheral capillary oxygen saturation.

^g^SpO2r: peripheral capillary oxygen saturation right.

^h^SpO2l: peripheral capillary oxygen saturation left.

^i^NBP: noninvasive blood pressure.

^j^NBPm: noninvasive blood pressure mean.

^k^NBPs: noninvasive blood pressure systolic.

^l^NBPd: noninvasive blood pressure diastolic.

^m^AFIB: atrial fibrillation.

^n^End AFIB alarm indicates the end of the AFIB status.

^o^Tachy: tachycardia.

^p^Tachy/p: tachycardia p wave.

^q^Vent: ventricular.

#### Narrative Data

In a previous publication, we reported detailed analysis of the narrative data provided by the 39 transplant/cardiac ICU nurses who responded to the preintervention survey [16]. Categories and themes identified in that report were related to (1) constant nuisance alarms and their effect on patient safety, (2) poor usability and complexity of medical devices, (3) the look-alike and sound-alike alarms, (4) the lack of support to the use of monitor watchers or integration of alarms into nursing call systems, and (5) unit-related factors to alarm management. The latter includes absence of policies and procedures on alarm management, the fact that unit layout may hinder response to alarms specifically when a nurse is assigned to patients who are far apart, and the need for further training on the cardiac monitors.

In the postintervention survey, 10 out of 24 (42%) nurses provided comments. These comments were matched for the preproject periods and were analyzed. Issues identified were very similar to our previous report [16] with a major focus on (1) the usability of the cardiac monitors and (2) the frequent alarms. In the postproject survey, nurses listed new cardiac monitor usability-related issues, such as the inability of the cardiac monitor to interpret ECG and nurses' inability to enter the “do not resuscitate” orders.

#### Importance of Alarm Issues Related to Cardiac Monitors

The respondents’ rankings of the nine statements about the importance of alarm issues specific to cardiac monitors (section 3 in the postintervention survey) is presented in Table 4. Frequent false alarms, difficulty in understanding alarm priority, and noise competition from nonclinical devices were ranked as the top three important issues interfering with alarm recognition and response in the two project periods. Difficulty in setting alarms properly because of lack of knowledge on the appropriate limits remained one of the least important issues in the postproject period. However, the lack of training on alarm systems rose from level 8 in the preintervention survey to level 4 in the postintervention survey. No significant differences were found in mean scores of the rankings between the preproject and postproject periods.

**Table 3:** Number and percentage of nurses who *agreed* or *strongly agreed* on the statements between the preproject and postproject periods (n=24).

Item	Statement^a^	Preproject, n (%)	Postproject, n (%)	% change^b^
1	Nuisance alarms occur frequently	24 (100)	18 (75)	-25.0
2	Nuisance alarms disrupt patient care	23 (96)	23 (96)	0
3	Nuisance alarms reduce trust in alarms and cause caregivers to inappropriately turn alarms off at times other than setup or procedural events	21 (88)	22 (92)	4.8
4	When a number of devices are used with a patient, it can be confusing to determine which device is in an alarm condition	21 (88)	19 (79)	-9.5
5	Smart alarms (eg, where multiple parameters, rate of change of parameters, and signal quality are automatically assessed in their entirety) would be effective to use for improving clinical response to important patient alarms	20 (83)	17 (71)	-15.0
6	Central alarm management staff responsible for receiving alarm messages and alerting appropriate staff is helpful	19 (79)	18 (75)	-5.3
7	Smart alarms (eg, where multiple parameters, rate of change of parameters, and signal quality are automatically assessed in their entirety) would be effective to use for reducing false alarms	19 (79)	16 (67)	-15.8
8^c^	Unit layout does interfere with alarm recognition and management	18 (75)	18 (75)	0
9	Alarm integration and communication systems via pagers, cell phones, and other wireless devices are useful for improving alarms management and response	15 (63)	17 (71)	13.3
10^c^	Nearly all alarms are actionable (requiring the nurse to respond and take an action)	14 (58)	14 (58)	0
11	Alarm sounds and/or visual displays of the current monitoring systems and devices should clearly differentiate the priority of alarm	13 (54)	14 (58)	7.7
12	Properly setting alarm parameters and alerts is overly complex in existing devices	13 (54)	13 (54)	0
13	Clinical staff is sensitive to alarms and responds quickly	13 (54)	15 (63)	15.4
14^c^	When a lethal alarm sounds, it is clearly and quickly recognized and immediate action is taken to address the alarm	12 (50)	14 (58)	16.7
15	Environmental background noise has interfered with alarm recognition	12 (50)	15 (63)	25.0
16	Alarm sounds and/or visual displays should be distinct based on the parameter or source (eg, device)	12 (50)	16 (67)	33.3
17^d^	There is a requirement in my unit to document that the alarms are set and are appropriate for each patient	11 (46)	18 (75)	63.6
18^d^	The alarms used on my unit are adequate to alert staff of potential or actual changes in a patient’s condition	10 (42)	9 (38)	-10.0
19	There have been frequent instances where alarms could not be heard and were missed	8 (33)	8 (33)	0
20^d^	The medical devices used on my unit all have distinct outputs (ie, sounds, repetition rates, visual displays) that allow users to identify the source of the alarm	8 (33)	15 (63)	87.5
21^d^	Clinical policies and procedures regarding alarm management are effectively used in my unit	6 (25)	11 (46)	83.3
22	Newer monitoring systems (eg, < 3 years old) have solved most of the previous problems we experienced with clinical alarms	1 (4)	6 (25)	500

^a^Edited and used with permission from the Healthcare Technology Foundation (HTF) 2011.

^b^Percent change = ((y2 - y1) / y1) × 100.

^c^These are the new statements that we added to our survey. They do not exist in the original HTF survey.

^d^These are the statements where the “floor/area of the hospital” or “institution” in the HTF clinical alarms survey were replaced with “unit” in our survey.

**Table 4:** Importance of alarm issues related to the cardiac monitors (n=24).

Item	Statement	Preproject		Postproject		
		Item response, mean	Mean ranking^a^	Item response, mean	Mean ranking	*P*
1^b^	Frequent false alarms, which lead to reduced attention or response to alarms when they occur	2.40	1	3.40	1	.11
2^b^	Difficulty in understanding the priority of an alarm	3.00	2	4.32	2	.07
3^b^	Noise competition from nonclinical alarms and pages	3.95	3	4.55	3	.50
4^c^	Lack of available policy on appropriate alarm parameters for individualized patients	4.40	4	5.80	9	.08
5^c^	The need to frequently reset alarm settings every time they revert back to default when the monitor is disconnected from the patient	4.42	5	5.16	5	.24
6^d^	Difficulty in hearing alarms when they occur, especially from outside patient room	4.47	6	5.37	7	.36
7^d^	Difficulty in setting alarms properly because of the complexity of the monitor	4.84	7	5.21	6	.70
8^b^	Lack of training on alarm systems	4.90	8	4.70	4	.83
9^d^	Difficulty in setting alarms properly because of lack of knowledge on the appropriate limits for my patient condition	5.42	9	5.58	8	.75

^a^Item response means were ranked from 1 (most important) to 9 (least important).

^b^These statements were adopted from the Healthcare Technology Foundation (HTF) survey.

^c^These statements were added to the survey to reflect the cardiac monitors.

^d^These statements were modified from the HTF survey. Original statements were as follows: item 6 “Difficulty in hearing alarms when they occur”; items 7 and 9 “Difficulty in setting alarms properly.”

### Practices Related to Clinical Alarms and Training on Cardiac Monitors

**Figure 1:**
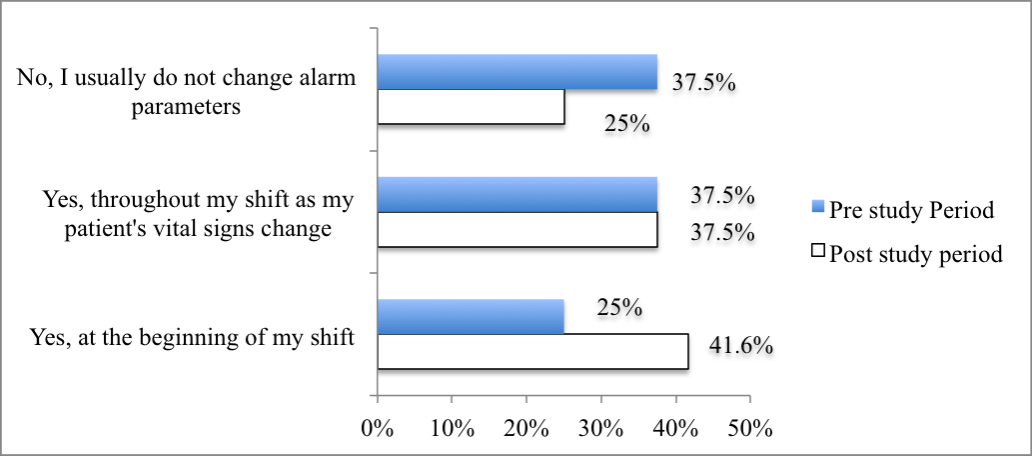
Percentage of nurses who modify the bedside alarm parameters in the pre- and postproject periods (n=24).

The data showed great variation among nurses in terms of changing alarm parameters (see Figure 1). More than one-third of nurses reported not adjusting alarm parameters in the preproject period. Despite the in-service, 25% (6/24) of nurses sustained the same practice in the postproject period. Additionally, only 38% (9/24) of nurses individualized parameters based on the patient’s vital signs in the two project periods.

**Figure 2:**
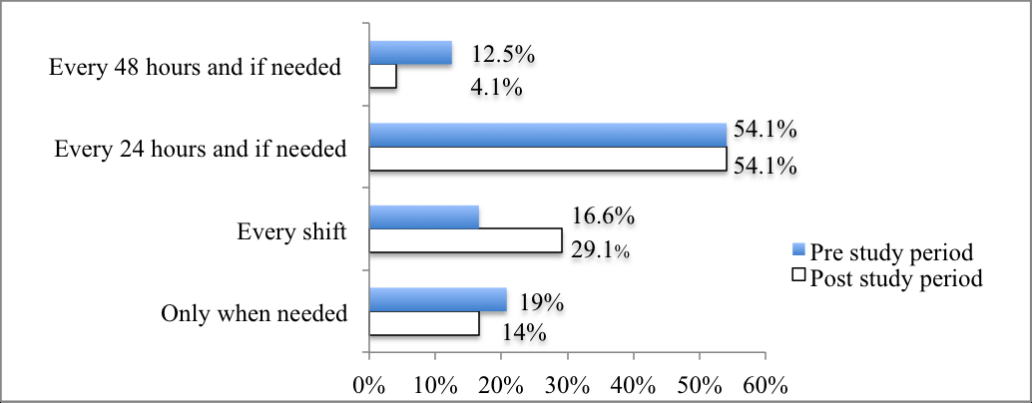
Percentage of nurses who replace patients' electrodes in the pre- and postproject periods (n=24).

The frequency of replacing patients' electrodes also varied. However, only 54% (13/24) of nurses changed them daily during the two project periods (see Figure 2).

**Figure 3:**
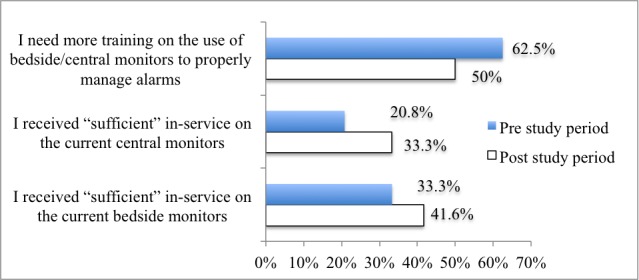
Percentage of nurses who received and needed monitor training in the pre- and postproject periods (n=24).

In regard to the training needed on the cardiac monitors, the majority of nurses indicated that they did not receive sufficient training on the central and bedside monitors (19/24, 79% and 16/24, 67%, respectively) in the preproject period. Despite the in-service, almost half of the nurses specified their need for more training in the postproject period (see Figure 3).

## Discussion

### Overview

Examining the effect of interventions targeting alarm systems safety on nurses' attitudes toward alarms and alarm fatigue related-practices is critical to evaluate improvements in the safety of these systems. Our unit-wide changes in default alarm settings of cardiac monitors significantly reduced 24% of the total number of the target alarms. However, changing default alarm settings, the subsequent reduction in alarm rate, and the in-service education on alarm management were insufficient to improve nurses’ attitudes toward alarms, alarm fatigue, or maintaining best clinical practices.

### Principal Findings and Future Directions

Finding alarms for parameters that were turned off supports the fact that bedside nurses customize patient alarms. ABP and SpO2 alarms were among the highest in the preproject and postproject data. The specific types of alarms (eg, Arterial Blood Pressure Mean [ABPm] and Arterial Blood Pressure Systolic [ABPs] alarms; see Table 2) can guide future initiatives on further alarm reduction. Future studies may examine if all specific types of alarms were necessary to be monitored for the patient. This may reveal alarm overuse and explain the high number of alarms. For example, clinicians need to determine if there is a need to monitor SpO2 right (SpO2r) and SpO2 left (SpO2l) for every patient.

The 65% increase in PVCs/min alarms (from 3233 to 5330) is expected because we tightened the parameter. However, the 41% increase in ST alarms (from 1852 to 2609) was unexpected given that we disabled this parameter. Changing this parameter to “On” by nurses is a plausible interpretation for such an increase. The ST parameter includes 12 leads. It would be helpful to analyze if nurses turned on the ST alarm as per the recommended cases by physicians and according to the suggested limits, which leads they adjusted, or if they overused the alarms. Correlating alarm rates and conditions to reliable monitoring conditions is critical and has not yet been investigated. For example, ST monitoring is not recommended in cases when arrhythmias such as atrial flutter and fibrillation are present or if the patient is continuously ventricularly paced. These cases will result in frequent false nonactionable ST alarms.

The unexpected increase in Asystole alarms (from 323 to 565, 75%) can be related to acuity of patients’ conditions and infrequent electrode placement. Another possible explanation from our observation is not adjusting the Pace Mode to “On” in the monitor for patients with temporary pacemakers who keep alarming Asystole. Our results also showed that nurses do not follow the unit protocol (ie, every 24 hours and if needed) when changing leads; enforcement of this policy should take place [11]. In a telemetry unit, proper skin preparation and electrode placement resulted in a significant reduction of ECG alarms [17].

Despite the significant reduction in alarm rate, key issues causing alarm fatigue and reducing trust in alarm systems according to nurses were the high frequency of nuisance alarms, the confusion in locating the alarming device, a unit layout that hinders alarm response, the inadequacy of alarm systems to alert nurses of changes in patients' conditions, the lack of clinical policies and procedures on alarm management, and the complexity of the newer monitoring systems. These multiple issues emphasize the fact that alarm management is very complex in ICUs. On the other hand, and similar to our previous results [16], the narrative data attributed nurses’ frustration and desensitization to alarms to poor usability of the cardiac monitoring systems.

It seems that the complexity of these monitors require interactive, well-designed, and periodic training. Our in-service, though individualized and focused on changing and individualizing alarm parameters and troubleshooting common problems, was insufficient to enhance appropriate monitor use. This is supported by finding that 50% of nurses believed they still needed training on cardiac monitor use and suggests (1) the need for usability testing of cardiac monitors, (2) the use of super-users, and (3) a competency checklist that includes key features for monitor use. Usability studies may reveal the complexity of the monitors, lack of knowledge about some features, or inappropriate use of the monitors. Studies supported the lack of clinicians’ awareness about, and understanding of, the complexity of cardiac monitors [16,18]. On the other hand, the wide variations in nurses’ practices and lack of adherence to protocols related to frequency of changing patients’ electrodes and parameters are major factors behind frequent nuisance alarms. Best practices should be enforced through unit policies. Inconsistent practices are indicative of the need for further education on appropriate programming and use of monitoring devices.

### Summary

Cardiac monitors are receiving increased attention in ICUs because of the high number of alarms triggered by these devices compared to other alarm-equipped ICU devices (ie, infusion pumps, dialysis pumps, and mechanical ventilators) [19,20]. Unlike other studies [12,13], our multimethod approach in addressing alarm fatigue was unsuccessful in improving attitudes toward alarms and safety practices. This can be related to the difference in patient population, the type and complexity of cardiac monitors in use, and nurses' noncompliance to best practices related to a lack of unit policies on alarm management. Inconsistent practices related to alarm management by medical, surgical, and ICU nurses have been reported [16,21]. Studies also support the perceived relationships between inappropriate setting of alarm parameters and the high number of false alarms in ICUs [22]. On the other hand, unlike many other observation-based clinical alarms safety studies [23,24], we measured alarm events using an objective data source of the audit log. The audit log provides a comprehensive record of all cardiac monitor alarms, except for the NBP Done Tone.

If we included the alarms from the monitor missed from the unit change and the NBP Done Tone alarms, the actual alarms’ reduction rate would be more than 24%. The inconsistency in applying the same unit of analysis in measuring alarm rates hinders further comparison across alarm safety studies.

### Limitations

The sample of nurses was small. This limited examining the statistical difference in attitudes toward clinical alarms. Although we achieved a significant reduction in alarm rate, we did not correlate that to the acuity of patient conditions preintervention and postintervention. Our description of alarms was limited to the alarms that we targeted for change. The audit log of the cardiac monitors records all types of physiologic alarms and all technical alarms. Analyzing other alarms may provide more insight into the total number of alarms triggered by the cardiac monitors. Our study was limited to alarms from the cardiac monitors and did not include other frequently used alarming devices in ICUs, such as infusion pumps or ventilators. However, cardiac monitors were the devices associated with the highest number of death cases in the US Food and Drug Administration data [19].

### Conclusions

Changing default alarm settings and standard in-service education on cardiac monitor use are insufficient to improve alarm systems safety. Alarm management in ICUs is very complex, involving alarm management practices by clinicians, availability of unit policies and procedures, unit layout, complexity and usability of monitoring devices, and adequacy of training on systems use. The complexity of the newer monitoring systems requires urgent usability testing. Multidimensional interventions are needed to improve alarm systems safety and attain the Joint Commission National Patient Safety Goal on alarm systems safety in critical care units.

## Appendix

Multimedia Appendix 1 Definitions of alarm events for parameters involved in the change.

## Abbreviations

ABP: arterial blood pressure
ABPm: arterial blood pressure mean
ABPs: arterial blood pressure systolic
AFIB: atrial fibrillation
ECG: electrocardiogram
HR: heart rate
HTF: Healthcare Technology Foundation
ICU: intensive care unit
IRB: Institutional Review Board
JC: Joint Commission
NBP: noninvasive blood pressure
NBPd: noninvasive blood pressure diastolic
NBPm: noninvasive blood pressure mean
NBPs: noninvasive blood pressure systolic
PVC: premature ventricular contraction
SpO2: peripheral capillary oxygen saturation
SpO2l: peripheral capillary oxygen saturation left
SpO2r: peripheral capillary oxygen saturation right
ST: ST segment in the electrocardiogram
Tachy: tachycardia
Tachy/p: tachycardia p wave
Vent: ventricular
