# Supplementary material for: Changes in Default Alarm Settings and Standard In-Service are Insufficient to Improve Alarm Fatigue in an Intensive Care Unit: A Pilot Project
Source: JMIR Hum Factors. 2016 Jan 11;3(1):e1. doi: 10.2196/humanfactors.5098 (PMC4797663; doi:10.2196/humanfactors.5098)
Supplement: Multimedia Appendix 1 [file humanfactors_v3i1e1_app1.pdf]

Alarm	Definition
Premature ventricular contractions per minute (PVCs/min)	A yellow (non-critical, moderate severity) Electrocardiogram (ECG)/arrhythmia alarm, signals when the number of PVCs within one minute exceeds the programmed limit of PVCs/min.
TachyClamp	Is the limit of a heart rate (HR) that if exceeded will announce a red (life-threatening) priority alarm requiring immediate clinician action, regardless of the programmed Upper HR limit. For example, if a nurse adjusted the Upper HR limit to 190 bpm (beat per minute) and TachyClamp was defaulted as 180 bpm, a patient HR of 181 bpm or higher will announce a TachyClamp red alarm while a HR of 191 bpm or higher will announce a yellow High HR alarm.
ExtremeTachy	Is a red ECG alarm, announced if a patient HR is XX bpm above the Upper HR limit, where XX is the default ExtremeTachy limit. For example, if the programmed Upper HR limit is 160 bpm and the default ExtremeTachy limit is 40 bpm, an ExtremeTachy will be announced for a patient with a HR of 200 bpm or higher.
Arterial blood pressure (ABP)	An invasive pressure yellow alarm. It has three sets (systolic, diastolic and mean) each with high and low limits. It alarms when the ABP is greater than the upper or lower than the lower programmed limits.
Pressure disconnect	This alarm signals only for the different types of arterial pressures in case of non-pulsatile blood pressure (no waveforms) “and when mean pressure is continuously less than 10mmhg” [15, page B-8].
Non-invasive blood pressure (NBP)	Is a yellow alarm with three sets (systolic, diastolic and mean) each with high and low limits. It alarms when NBP is greater than the upper or lower than the lower programmed limits.
System pulse	Is one of the yellow ECG monitoring parameter that measures the average pulse rate over 1 min and alarms if greater than the upper or lower than the lower programmed limits.
Asystole	Is a red ECG alarm, signals when no QRS is detected for a period greater than the default asystole time (2.5 or 4 seconds).
Peripheral capillary	Is a yellow alarm captured by the pulse oximetry. The monitor will alarm if the

oxygen saturation (SpO2)	SpO2 value exceeds the upper or is lower than the lower programmed limits.
St-Analysis	ST can be analyzed for 12 leads and alarms (yellow alarm) when an ST lead is greater than the upper limit or lower than the lower limit.
Run PVCs	Is a yellow ECG/arrhythmia alarm, signals when there is “a run of PVCs greater than 2” [15, page B-4].
Pair PVCs	A yellow ECG/arrhythmia alarm of two successive (PVCs) between non-PVCs.
Vent Bigeminy	A yellow ECG/arrhythmia alarm of “a dominant rhythm of N, V, N, V, N. N= supraventricular beats, V=ventricular beat” [15, page B-4].
Vent Trigeminy	A yellow ECG/arrhythmia alarm of “a dominant rhythm of N, N, V, N, N, V, N, N. N= supraventricular beats, V=ventricular beat” [15, page B-4].
Multiform PVCs	A yellow ECG/arrhythmia alarm. “The occurrence of two differently shaped Vs, each occurring at least twice within the last 300 beats as well as each occurring at least once within the last 60 beats” [15, page B-4].
Pause	A yellow ECG/arrhythmia alarm of no heart beat (QRS) detected for a period greater than the programed pause time.
Missed beats	A yellow ECG/arrhythmia alarm of “no beat detected for 1.75 X average R-R interval for HR < 120, or no beat for 1 second with HR > 120 (non-paced patient only)” [15, page B-4].
Atrial fibrillation (AFIB)	A yellow ECG/arrhythmia alarm signals when an irregular heart rate (irregular RR intervals) of AFIB was detected.
End AFIB	A yellow ECG/arrhythmia alarm that signals after the recovery from the atrial fibrillation condition for the programmed period after the first detection [15, page B-4].
NBP Done-Tone	A seemingly non-actionable excessive audible alarm especially when the NBP is cycling every 15 minutes and is announced automatically by the monitor after measuring the patient blood pressure.
